# Pelvic organ prolapse surgery and overactive bladder symptoms—a population-based cohort (FINPOP)

**DOI:** 10.1007/s00192-021-04920-w

**Published:** 2021-07-10

**Authors:** Päivi K. Karjalainen, Anna-Maija Tolppanen, Nina K. Mattsson, Olga A.E. Wihersaari, Jyrki T. Jalkanen, Kari Nieminen

**Affiliations:** 1 Department of Obstetrics and Gynecology, Hospital Nova of Central Finland, Hoitajantie 3, 40620 Jyväskylä, Finland; 2 Institute of Clinical Medicine, University of Eastern Finland, Kuopio, Finland; 3 School of Pharmacy, University of Eastern Finland, Kuopio, Finland; 4 Department of Obstetrics and Gynecology, Kanta-Häme Central Hospital, Hämeenlinna, Finland; 5 Department of Obstetrics and Gynecology, Tampere University Hospital, Tampere, Finland; 6 Faculty of Medicine and Health Technology, Tampere University, Tampere, Finland; 7 Central Finland Health Care District, Jyväskylä, Finland

**Keywords:** Overactive bladder, Pelvic organ prolapse, Pelvic organ prolapse surgery, Urgency urinary incontinence, Urinary frequency

## Abstract

**Introduction and hypothesis:**

It is unclear how compartment of pelvic organ prolapse (POP) impacts overactive bladder (OAB) symptom severity or improvement after POP surgery. We hypothesized that anterior and apical prolapse are more strongly associated with OAB symptoms than posterior compartment prolapse.

**Methods:**

A total of 2933 POP surgeries from a prospective population-based cohort were divided into two groups: (1) anterior and/or apical compartment surgery (± posterior repair), *N* = 2091; (2) posterior repair only, *N* = 478. Urinary frequency and urgency urinary incontinence (UUI) were evaluated using PFDI-20 (bothersome symptom: score 3–4) at baseline, 6, and 24 months. Association between degree of POP in specific compartments and symptoms at baseline was estimated with generalized linear models and between compartment of surgery and symptom improvement with generalized estimating equations.

**Results:**

At least one bothersome symptom was reported by 40% at baseline, 14% at 6, and 19% at 24 months. At baseline, urinary frequency was associated with degree of anterior and apical and UUI with anterior compartment prolapse. Women undergoing surgery for anterior/apical compartment started with worse symptoms and experienced greater improvement than women undergoing posterior compartment surgery. Bothersome frequency resolved in 82% after anterior/apical and in 63% after posterior compartment surgery. Bothersome UUI resolved in 75% after anterior/apical and in 61% after posterior compartment surgery. After surgery, symptom severity was comparable between groups. Bothersome de novo symptoms occurred in 1–3%.

**Conclusions:**

OAB symptoms are more strongly related to anterior and apical than to posterior compartment prolapse, but improvement is seen after surgery for any vaginal compartment.

## Introduction

Overactive bladder (OAB) symptoms are common, affecting around 13% of women of all ages. The prevalence of these symptoms increases with age, and they can have a detrimental impact on the quality of life [1]. Community-based studies show that OAB symptoms are up to six times more frequent among women with pelvic organ prolapse (POP) compared with age-adjusted women without POP [2]. OAB symptoms also improve after POP surgery, implying a connection between the two [2]. Nevertheless, the role of POP as an explanatory pathology behind OAB remains unclear, and current guidelines do not list POP in the diagnostic algorithms for OAB [3, 4].

Proposed mechanisms to explain the co-occurrence of OAB symptoms and POP include detrusor overactivity due to (1) bladder outflow obstruction, (2) bladder wall distension and stimulation of stretch receptors, and (3) traction and opening of the urethra triggering the emptying reflex [2]. Based on these theories, it is plausible that anterior compartment prolapse (i.e., bladder involvement) has a greater impact on the OAB symptoms than posterior compartment prolapse. However, the majority of studies have not found any correlation among the degree of anterior, apical, or posterior compartment prolapse and severity of OAB symptoms [2]. Furthermore, studies comparing symptom improvement after prolapse surgery for different vaginal compartments conclude comparable improvement regardless of the repaired compartment [5–8]. This lack of association, together with imperfect symptom resolution, challenges the rationale to perform POP surgery to address OAB symptoms [9, 10].

To understand the relationship between OAB symptoms and POP, we [1] quantified the association between the degree of individual POP compartments and OAB symptoms before surgery and [2] examined whether symptom improvement after surgery is dependent on the repaired compartment. We hypothesized that OAB symptoms are more strongly related to the anterior and apical than to the posterior compartment prolapse.

## Materials and methods

### Setting and participants

We used data from the national, prospective Finnish Pelvic Organ Prolapse Surgery Survey (FINPOP). The study setting, population, and methods of surgery have been reported in more detail previously [11]. All Finnish hospitals performing POP surgery were invited to participate and to recruit all patients scheduled to undergo prolapse surgery during 2015. Women unable to communicate in Finnish or Swedish were excluded. ﻿A total of 41 of 45 hospitals (91%) performing POP surgery participated. The FINPOP cohort includes 3535 POP operations representing 83% of POP operations performed nationwide during 2015 (﻿National database: Care Register for Health Care).

**Fig. 1:**
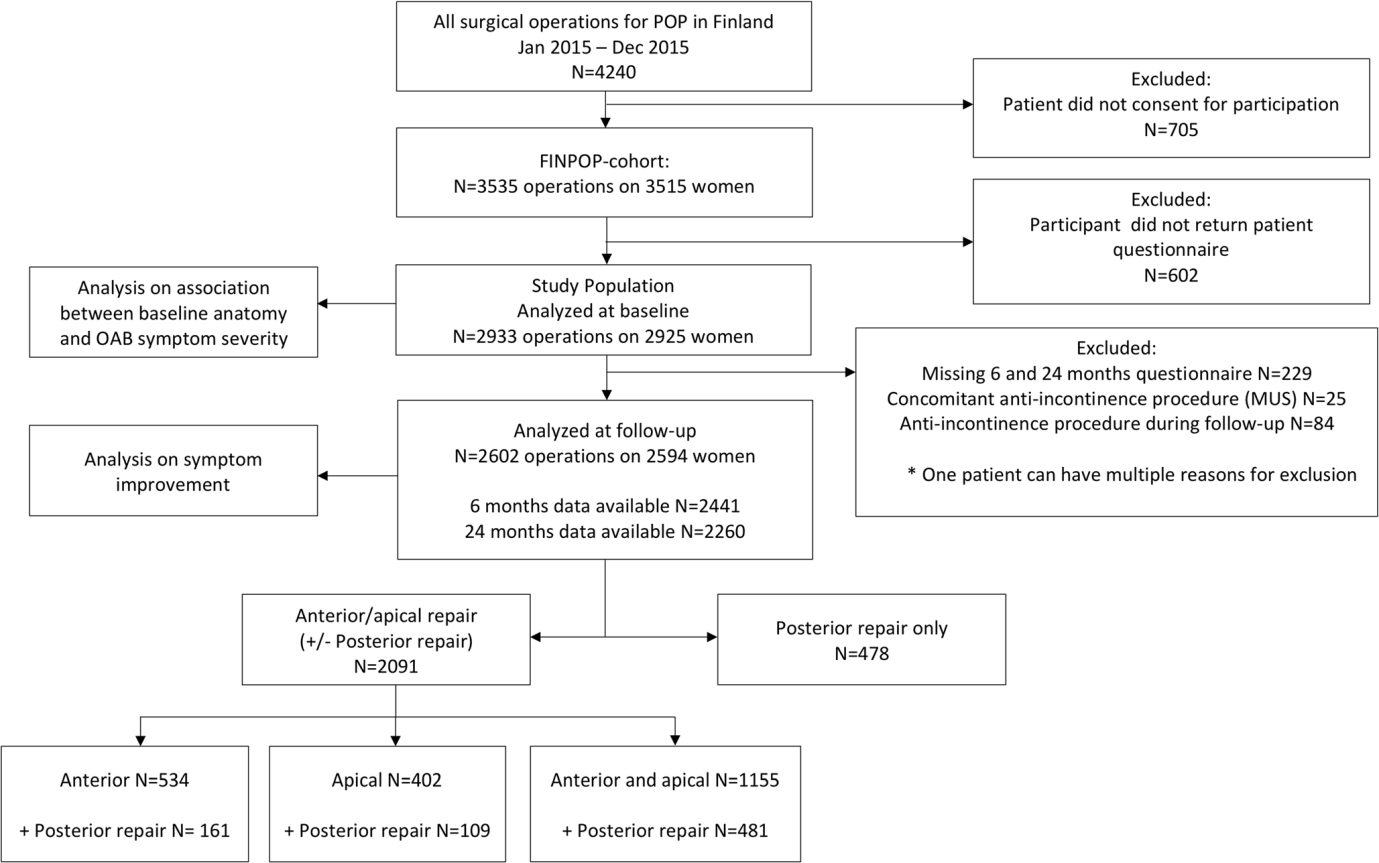
Flowchart showing the selection of the study population. POP, pelvic organ prolapse; FINPOP, Finnish Pelvic Organ Prolapse Surgery Survey

The population of this study includes 2933 operations with preoperative clinical examination and symptom questionnaires available. The patient flow, exclusion criteria, and data availability are shown in Fig. 1. We excluded women receiving a procedure for stress urinary incontinence concomitantly (*N* = 25) or during the follow-up (*N* = 84) from the analyses regarding symptom improvement. Of six women receiving intradetrusor injections of botulinum toxin A between 6 and 24 months’ follow-up, two were excluded from 24 months’ analyses because they reported improvement in the OAB symptom scores. Since usage of OAB medication at baseline did not associate with fewer OAB symptoms (rather the opposite), we did not exclude these women from the analyses.

### Data collection

All data in this study were collected prospectively, and information was not retrieved from hospital charts.

The preoperative degree of POP was assessed by the surgeons using the simplified Pelvic Organ Prolapse Quantification (POP-Q) system as a single most distal point (in centimeters from the hymen) for each vaginal compartment (Ba for anterior; Bp for posterior, and C for apical compartment). The stage of POP was determined according to the POP-Q system [12]. Vaginal length was not recorded, and therefore stages 3–4 for all compartments, as well as stages 0–1 for apical prolapse, were combined in the analyses. The surgeons also recorded participants’ surgical history and details on the operation performed. The surgeons entered the data in the electronic study registry in a standardized form.

The participants completed standardized, self-administered questionnaires at baseline and at 6 and 24 months after the surgery. This included information on their medical, surgical, and obstetric history. Pelvic floor dysfunction was assessed with a validated, condition-specific quality-of-life instrument, Pelvic Floor Distress Inventory −20 (PFDI-20) [13, 14]. The follow-up questionnaires were mailed to the participants, collected on paper or electronic forms based on participant’s preference, and entered in the electronic study registry.

Information on the anti-incontinence procedures during the follow-up were retrieved from the Care Register for Health Care (coverage > 95%) [15].

### Outcome measures

OAB symptoms were evaluated using two items in the PFDI-20: Item 15, ‘Do you usually experience frequent urination?’, assessed urinary frequency; item 16, ‘Do you usually experience urine leakage associated with a feeling of urgency, that is, a strong sensation of needing to go to the bathroom?’, assessed urgency urinary incontinence [14]. The scale for each symptom is as follows: 0: symptom not present; 1: symptom present but not at all bothersome; 2: symptom somewhat bothersome; 3: symptom moderately bothersome; 4: symptom quite a bit bothersome. We defined answers 3 and 4 as bothersome symptoms. Bothersome symptom was defined as resolved when bother score at follow-up was < 3.

### Statistical analyses

We categorized the population into two groups based on the repaired compartment: (1) women who had surgery for anterior and/or apical compartment (± posterior compartment), i.e., anterior/apical group, and (2) women who had surgery for posterior compartment only, i.e., posterior group.

To further explore differences between anterior and apical repairs, we performed a secondary analysis dividing the anterior/apical group into women with (1) a vaginal procedure for anterior wall but not any type of apical procedure (anterior group); (2) any type of apical procedure but no vaginal procedure for anterior vaginal wall (apical group); (3) a vaginal procedure for anterior wall and any type of apical procedure (anterior and apical group).

We used a generalized linear model (ordinal logistic) to estimate the association between the baseline anatomy (Ba, Bp, C in centimeters) and symptom bother (ordinal scale 0 to 4). Multivariable models were fitted to control for prolapse in other compartments (Ba, Bp, C) and to adjust for potential confounders. The confounders (age, BMI, parity, smoking, previous POP surgery, previous anti-incontinence surgery) were selected based on the knowledge from previous literature and from clinical experience using directed acyclic graphs [16]. Spearman’s correlation coefficients did not indicate strong collinearity between the variables (all < 0.4). The ordinal logistic model yields odds ratios (OR) with 95% confidence intervals (CI) for a higher bother score with a centimeter increase in the extent of prolapse.

To estimate the association between the site/compartment of surgery and improvement after surgery, we used ordinal generalized estimation equations adjusting for confounders. To assess whether anterior/apical repair improved symptoms more compared with posterior repair, time * repair group interaction was included in the model. We also performed a sensitivity analysis adjusting for concomitant posterior repair.

Estimated marginal means from separate models with continuous dependent variables (instead of ordinal) were used to plot graphs to illustrate the results.

### Ethical aspects

The study followed the ethical standards for human experimentation established by the Declaration of Helsinki of 1964, revised in 2013. The Research Ethics Committee of the Northern Savo Hospital District approved the study on May 20, 2014 (reference number 5//2014), and each participating hospital granted an approval for conducting the study. All participants gave written consent.

## Results

### Characteristics of the study population

The characteristics of the study population (*N* = 2933) are presented in Table 1. Of women with follow-up data (*N* = 2602), 2091 (81%) underwent surgery for the anterior and/or apical (± posterior) compartment and 478 (19%) for the posterior compartment only (Fig. 1). One hundred fifty-seven (6%) women self-reported a re-operation for any recurrent prolapse during the 2-year follow-up.

**Table 1:** Characteristics of the study population

Characteristic	Study population *N* = 2933	Data missing *n* (%)
Age (years), mean ± SD	64.0 ± 10.5	2 (0.1)
BMI (kg/m²), mean ± SD	26.9 ± 4.1	99 (3.4)
Parity, median (IQR)	2 (1)	47 (1.6)
Current smoker, *n* (%)	255 (8.7)	11 (0.4)
Diabetes, *n* (%)	283 (9.6)	0
Prior hysterectomy, *n* (%)	981 (33.4)	0
Prior POP surgery, *n* (%)	740 (25.2)	0
Prior anti-incontinence surgery, *n* (%)	170 (5.8)	0
POP-Q point Ba ≥ 0, *n* (%)	1859 (65.5)	96 (3.3)
POP-Q point C ≥ 0, *n* (%)	1138 (40.6)	130 (4.4)
POP-Q point Bp ≥ 0, *n* (%)	1259 (44.5)	105 (3.6)
PFDI-20 score, mean ± SD	99.1 ± 49.9	5 (0.2)
OAB medication, *n* (%)	97 (3.3)	0
Local or systemic estrogen therapy, *n* (%)	2429 (82.9)	4 (0.1)
Type of surgery, *n* (%)		0
Native tissue repair	2357 (80.4)	
Vaginal mesh	362 (12.3)	
Abdominal mesh	214 (7.3)	

SD, standard deviation; BMI, body mass index; IQR, interquartile range; POP, pelvic organ prolapse; POP-Q, Pelvic Organ Prolapse Quantification System; PFDI-20, Pelvic Floor Distress Inventory-20; OAB, overactive bladder

### Prevalence of OAB symptoms at baseline

At baseline, 2346 women (82%) reported at least one OAB symptom of any degree and 1135 (40%) at least one bothersome (bother score 3 or 4) OAB symptom (Table 2). Altogether 1303 (46%) women presented with both urinary frequency and urgency urinary incontinence of any degree, and 484 (17%) presented with bothersome urinary frequency and urgency urinary incontinence.

**Table 2:** Prevalence of OAB symptoms at baseline and at follow-up

Repair group	Symptom present	Urinary frequency			Urgency incontinence			Either symptom		
		Baseline	6 months	24 months	Baseline	6 months	24 months	Baseline	6 months	24 months
**All**	**Yes; any degree of bother**	1890 (66.9)	828 (33.7)	910 (39.6)	1759 (61.8)	1186 (48.2)	1158 (50.7)	2346 (82.3)	1422 (57.9)	1451 (63.4)
	**Yes, bothersome symptom**	875 (31.0)	204 (8.3)	258 (11.2)	744 (26.2)	257 (10.4)	326 (14.3)	1135 (40.4)	331 (13.6)	423 (18.6)
	**No**	935 (33.1)	1630 (66.3)	1390 (60.4)	1086 (38.2)	1274 (51.8)	1124 (49.3)	504 (17.7)	1035 (42.1)	838 (36.6)
**Anterior/Apical**	**Yes; any degree of bother**	1568 (69.8)	650 (33.0)	733 (39.8)	1462 (64.6)	958 (48.7)	947 (51.9)	1929 (85.1)	1136 (57.8)	1175 (64.1)
	**Yes, bothersome symptom**	735 (32.7)	159 (8.1)	198 (10.8)	618 (27.3)	202 (10.3)	261 (14.3)	945 (42.3)	255 (13.1)	333 (18.3)
	**No**	678 (30.2)	1321 (67.0)	1108 (60.2)	801 (35.4)	1011 (51.3)	878 (48.1)	338 (14.9)	831 (42.2)	657 (35.9)
**Posterior**	**Yes; any degree of bother**	297 (54.6)	165 (35.8)	164 (38.1)	273 (49.9)	213 (45.7)	197 (45.8)	387 (70.6)	268 (57.8)	260 (60.5)
	**Yes, bothersome symptom**	129 (23.7)	43 (9.3)	57 (13.2)	114 (20.8)	51 (10.9)	60 (14.0)	175 (32.4)	72 (15.7)	84 (19.7)
	**No**	247 (45.4)	296 (64.2)	267 (61.9)	274 (50.1)	253 (54.3)	233 (54.2)	161 (29.4)	196 (42.2)	170 (39.5)

Numbers presented as number (%) of data available at each time point

### Association between anatomy and OAB symptoms at baseline

**Fig. 2:**
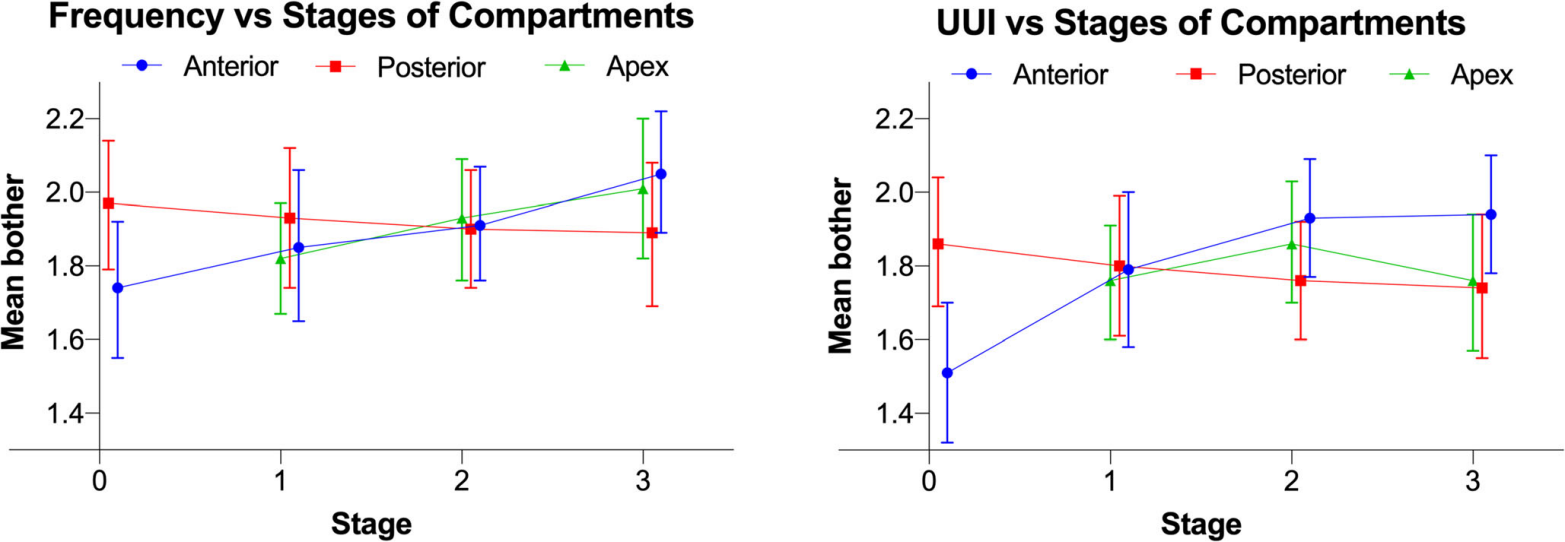
Association between the severity of overactive bladder symptoms and degree and compartment of prolapse at baseline. UUI, urgency urinary incontinence. Adjusted estimated marginal means with 95% confidence intervals for stages of individual compartments is shown. Stage 3 combines stages 3 and 4 for all compartments. For apical compartment, stage 1 combines stages 0 and 1

The severity of urinary frequency increased with advancing anterior wall and apical prolapse, while posterior wall prolapse was not associated with this symptom. The odds for a higher bother score increased by 7% (95% CI 3–11%) for anterior wall and by 4% (95% CI 1–6%) for apical prolapse per centimeter of additional descent (multivariable model) (Appendix Table 3, Fig. 2). The crude prevalence of bothersome urinary frequency increased from 26% (95% CI 22–31%) to 37% (95% CI 33–40%) and from 29% (95% CI 26–31%) to 36% (95% CI 31–40%) from stage 0 to stage 3–4 of anterior wall and from stage 0–1 to stage 3–4 of apical prolapse, respectively (Appendix Table 4).

The severity of urgency urinary incontinence increased with advancing anterior wall prolapse; the association with the posterior wall prolapse was inverse, and there was no significant association with apical prolapse. The odds for a higher bother score increased by 8% (95% CI 4–13%) for anterior wall and decreased by 4% (95% CI 1–8%) for posterior wall prolapse per centimeter of additional descend (multivariable model) (Appendix Table 3, Fig. 2). The crude prevalence of bothersome urgency urinary incontinence increased from 20% (95% CI 16–24%) for stage 0 to 29% (95% CI 26–32%) for stage 3–4 of anterior wall prolapse and decreased from 30% (95% CI 27–33%) for stage 0 to 26% (95% CI 22–30%) for stage 3–4 of posterior wall prolapse (Appendix Table 4).

### Symptom relief after surgery

**Fig. 3:**
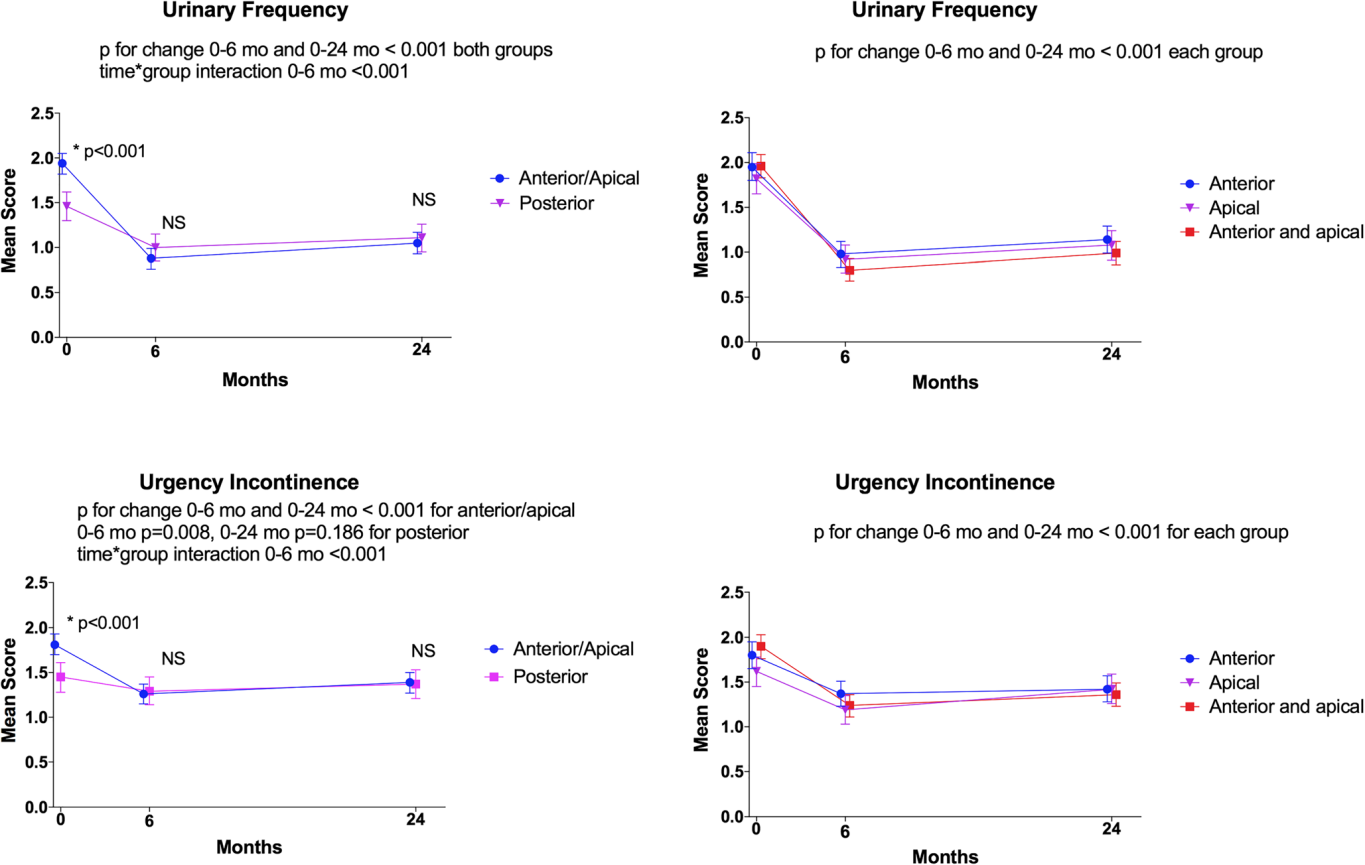
Impact of surgery on overactive bladder symptoms. Impact of prolapse surgery on the overactive bladder symptom severity during the follow-up is shown. On the Y-axis, estimated marginal means (and their 95% confidence intervals) from linear generalized estimating equations multivariable model (scale 0–4, higher number indicating higher symptom bother: 0: symptom not present, 1: symptom present but not at all bothersome; 2: symptom somewhat bothersome; 3: symptom moderately bothersome; 4: symptom quite a bit bothersome). On the X-axis, follow-up points. On the left column, data stratified into two surgical groups. On the right, anterior/apical group stratified into three groups. The asterisk indicates *P* < 0.05, and NS indicates not significant (*P* > 0.05) for between-group comparison in an ordinal logistic generalized estimated equations model at different time points. *P*-values for within-group improvement and time*group interaction are reported for ordinal models. Between-group comparisons performed only for two groups (i.e., anterior/apical vs. posterior)

The severity of urinary frequency and urgency urinary incontinence decreased after surgery for all compartments (anterior, apical, anterior and apical, posterior) during the 6-month follow-up (*p* < 0.008 for all). At 24 months, symptom severity remained better compared with the baseline except for urgency urinary incontinence among women undergoing posterior repair only (*p* = 0.186 for posterior group and < 0.001 for other groups) (Fig. 3).

Women undergoing anterior/apical compartment surgery had worse symptom severity at baseline (< 0.001) and experienced greater symptom improvement after surgery than women undergoing surgery for posterior compartment only (time*group interaction < 0.001). There was no difference in the symptom severity after the surgery between the two groups (*p* > 0.05 for all) (Fig. 3). A sensitivity analysis adjusting for concomitant posterior repair yielded similar results (data not shown).

The prevalence of bothersome urinary frequency and urgency urinary incontinence in the total population decreased from 0.31 to 0.08 [relative risk (RR) 0.27] and 0.26 to 0.10 (RR 0.40) during 6 months’ follow-up, respectively (Table 2).

**Fig. 4:**
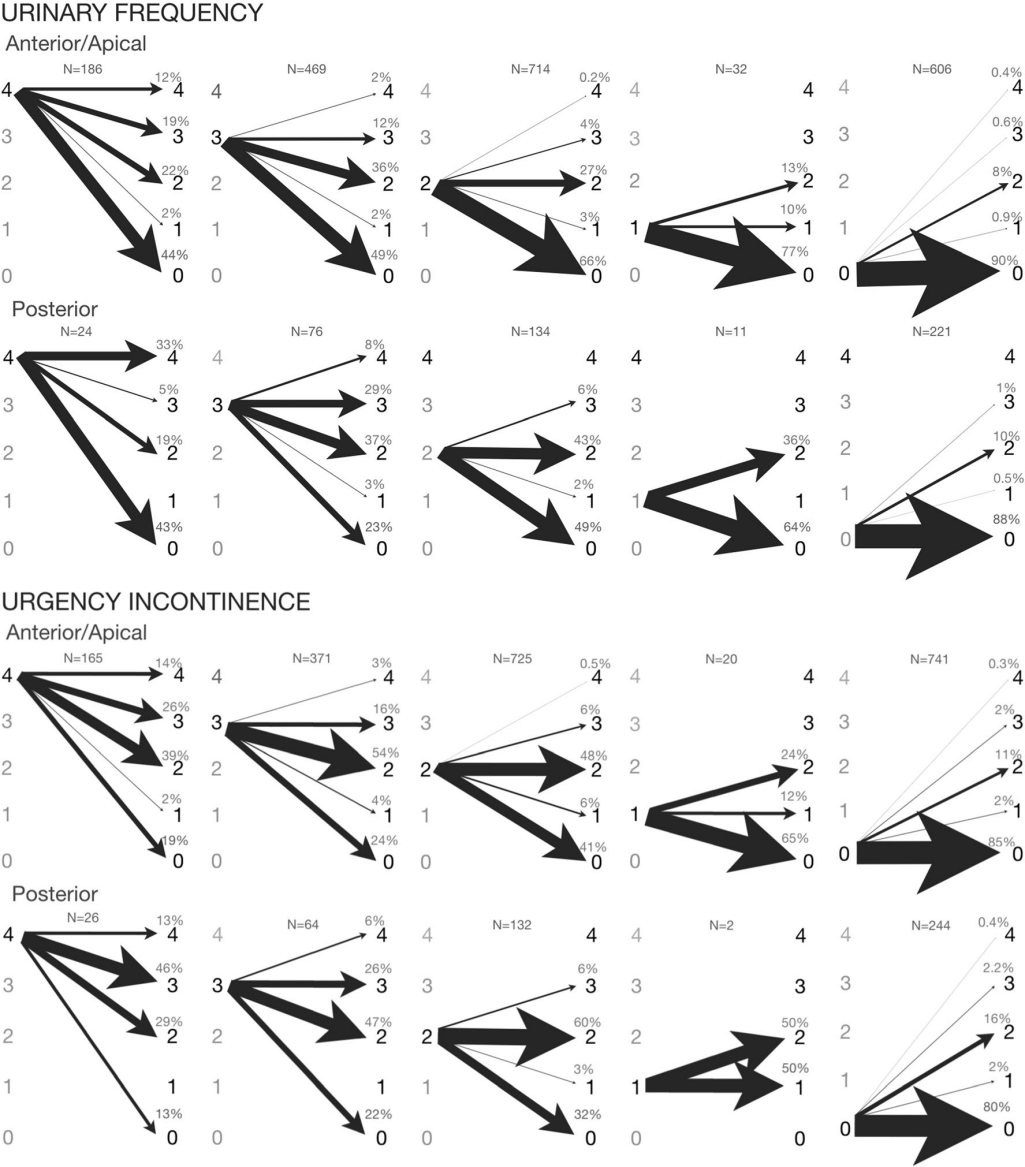
Change in symptom severity from baseline to 6 months for each overactive bladder symptom stratified by baseline symptom severity in the anterior/apical and the posterior groups. Each image depicts change in symptom severity from baseline to 6 month’s follow-up stratified by baseline symptom severity: baseline symptom severity on the left and 6 months’ symptom severity on the right. The scale of symptom severity: 4: symptom quite a bit bothersome; 3: symptom moderately bothersome; 2: symptom somewhat bothersome; 1: symptom present but not at all bothersome; 0: symptom not present. The thickness of the arrow is proportional to percentage

In the anterior/apical group, urinary frequency bother score improved in 994/1284 (77%) women at 6 months and in 861/1202 (72%) at 24 months. Bothersome urinary frequency resolved in 487/597 (82%) women at 6 and in 426/559 (76%) at 24 months. Women with bother score of 0 at baseline reported de novo urinary frequency of any degree in 54/560 (10%) and of bothersome degree in 5/560 (1%) at 6 months. Urgency urinary incontinence bother score had improved in 718/1166 (62%) women at 6 months and in 623/1089 (57%) at 24 months. Bothersome urgency urinary incontinence had resolved in 363/484 (75%) women at 6 and in 301/459 (66%) at 24 months. The risk of de novo urgency urinary incontinence of any degree was 103/685 (15%) and of bothersome degree 14/685 (2%) at 6 months (Fig. 4).

In the posterior group, urinary frequency bother score had improved in 122/216 (57%) women at 6 months and in 111/202 (55%) at 24 months. Bothersome urinary frequency had resolved in 52/83 (63%) women at 6 and in 44/76 (58%) at 24 months. Women with bother score of 0 at baseline reported de novo urinary frequency of any degree in 24/205 (12%) and of bothersome degree in 2/205 (1%) at 6 months. Urgency urinary incontinence bother score had improved in 99/197 (50%) women at 6 months and in 76/186 (41%) at 24 months. Bothersome urgency urinary incontinence had resolved in 48/79 (61%) women at 6 and 39/73 (53%) at 24 months. The risk of de novo urgency urinary incontinence of any degree was 47/229 (21%) and of bothersome degree 6/229 (3%) at 6 months (Fig. 4).

## Discussion

### Principal findings

OAB symptoms among women undergoing POP surgery are common (urinary frequency or urgency urinary incontinence of any and bothersome degree observed in 82% and 40% of women) and depend on the compartment and severity of prolapse. The symptoms had stronger associations with the anterior and apical compartment than posterior compartment prolapse, as hypothesized. Consistent with this finding, surgery for the anterior and/or apical compartment resulted in greater postoperative symptom relief compared to posterior repair. After surgery, women reached a similar level of OAB symptoms regardless of the repaired compartment. At 6 months, 14% of women reported bothersome urinary frequency or urgency urinary incontinence.

The degree of prolapse explained only a small proportion of variation in the symptom severity at baseline (e.g., 11% absolute increase in the prevalence of bothersome urinary frequency from no anterior wall prolapse to stage 3–4). Nevertheless, the correction of anatomy relieved a significant proportion of OAB symptoms. Six months after anterior/apical compartment surgery, urinary frequency bother score had decreased in 77% and urgency urinary incontinence in 62% of women. Although the symptoms did not correlate with the degree of posterior wall prolapse at baseline, OAB symptoms’ bother score still decreased after posterior repair in 50-57% of women. Bothersome de novo symptoms were uncommon (1–3% 6 months postoperatively).

### Results in the context of what is known

POP and OAB symptoms often coexist, but evidence on the correlation between the specific anatomical defect and symptom severity is conflicting. Results from the majority of studies do not support any correlation between the degree of anterior wall [17–24], posterior wall [19–22, 24], apical [20, 21, 24, 25] or overall [21] prolapse and OAB symptoms [2]. However, some studies report a correlation, and at least four studies agree with our findings reporting more OAB symptoms with advancing degree of anterior wall prolapse [25–28].

A systematic review concludes that OAB symptoms improve after POP surgery [2]. However, it has remained unclear how the improvement relates to surgery for specific vaginal compartments. We identified only four previous studies comparing anterior to posterior involvement, and these studies could not consistently demonstrate a difference between anterior and posterior compartment surgery [5–8].

The reason for the conflicting results may lie in the study populations and methods: small sample size [17–21, 23], lack of contrast due to insufficient numbers of women with small [18, 19] or advanced [21] POP, and dichotomization of variables [5, 7, 19, 21–24] all reduce the ability to detect association [29]. Another common shortcoming is that the analyses are not controlled for prolapse in other compartments [19–23, 28]. This is essential as different anatomic defects likely contribute to different kind of pelvic floor dysfunctions.

Weak association between the degree of prolapse and OAB symptoms as well as incomplete symptom improvement after surgery imply that other factors explain a large part of the variation in OAB symptoms among the POP population. OAB is a nonspecific, complex, and multifactorial symptom syndrome frequent in the general population, and among men as well [1, 2]. There appear to be several distinct subtypes of OAB with multiple different mechanisms. The underlying factors can overlap and have convoluted interactions. Several potential pathophysiological factors, including metabolic syndrome, affective disorders, gastrointestinal functional disorders, sex hormone deficiency, urinary microbiota, and subclinical autonomic nervous system dysfunctions, have been suggested [30]. Since surgery does not address these potential underlying causes, POP surgery will not lead to resolution of OAB symptoms in all cases.

### Clinical implications

Patients, as well as clinicians, often assume that POP causes their OAB symptoms. Our data show that while the symptoms are not explained solely by the distorted anatomy, women nevertheless have high probability of symptom improvement without any further intervention.

Based on our results, POP can be considered a contributing factor to OAB symptoms, and it should be evaluated for in the diagnostic workup of women with these symptoms.

### Strengths and limitations

To the best of our knowledge, this is the largest study on the topic. A national cohort including all levels of hospital care increases the generalizability of the results. Further strengths include prospective data collection and use of a validated questionnaire. Unlike previous studies [5–7], we excluded women with anti-incontinence procedures. This is important since anti-incontinence surgery may independently affect OAB symptoms [2]. We also analyzed the outcomes on the actual measurement scale instead of dichotomization.

Our study has limitations. The population is comprised of women scheduled for POP surgery. This may lead to referral bias overestimating the effect. Second, we did not use a specific scale for OAB or collect data on urinary urgency and nocturia, two additional symptoms of OAB. Third, we did not obtain frequency volume charts or urodynamic studies and lack objective measures of OAB. Fourth, we did not collect data on OAB medication at follow-up. Finally, the observational nature of the study precludes drawing a definite causal relationship between POP and OAB.

## Conclusion

Urinary frequency and urgency urinary incontinence are common among women with POP and more strongly related to anterior and apical than to posterior compartment prolapse. Substantial symptom improvement is seen after surgery for any vaginal compartment, and bothersome de novo symptoms are rare. Residual postoperative symptoms are likely explained by the multifactorial nature of OAB symptoms.

## Appendix

**Table 3:** Association between overactive bladder symptoms and individual vaginal compartments at baseline

Independent variable	Urinary frequency				Urgency urinary incontinence			
	OR^a^	95% CI	aOR^b^	95% CI	OR^a^	95% CI	aOR^b^	95% CI
Ba (anterior wall prolapse)	**1.11**	**1.07–1.15**	**1.07**	**1.03–1.11**	**1.11**	**1.07–1.14**	**1.08**	**1.04–1.13**
Bp (posterior wall prolapse)	0.97	0.93–1.00	0.99	0.95–1.03	**0.94**	**0.91–0.97**	**0.96**	**0.92–0.99**
C (apical prolapse)	**1.05**	**1.03–1.07**	**1.04**	**1.01–1.06**	1.04	1.00–1.05	1.01	0.98–1.03

OR, odds ratio; CI, confidence interval; aOR, adjusted odds ratio

^a^Generalized linear models, univariate analysis; ^b^generalized linear models, multivariable model. Adjusted for prolapse in other compartments, age, BMI, parity, smoking, history of pelvic organ prolapse and anti-incontinence surgery

**Table 4:** Prevalence of overactive bladder symptoms at baseline per stages of individual compartments

Symptom	Compartment	Stage	Any degree of bother^a^		Bothersome symptom^b^	
			n with symptom/n total	% (95% CI)	n with symptom/n total	% (95% CI)
Frequency	Anterior	0	264/444	59.5 (54.7–64.1)	116/444	26.1 (22.1–30.5)
		1	184/298	61.7 (56.0–67.3)	85/298	28.5 (23.5–34.0)
		2	785/1157	67.8 (65.1–70.5)	349/1157	30.2 (27.5–32.9)
		3–4	602/831	72.4 (69.3–75.5)	303/831	36.5 (33.2–39.8)
	Posterior	0	492/700	70.3 (66.7–73.7)	231/700	33.0 (29.5–36.6)
		1	312/434	71.9 (67.4–76.1)	135/434	31.1 (26.8–35.7)
		2	759/1183	64.2 (61.4–66.9)	350/1183	29.6 (27.0–32.3)
		3–4	259/404	64.1 (59.2–68.8)	130/404	32.2 (27.6–37.0)
	Apex	0–1	858/1330	64.5 (61.9–67.1)	379/1330	28.5 (26.1–31.0)
		2	633/911	69.5 (66.4–72.5)	300/911	32.9 (29.9–36.1)
		3–4	324/456	71.1 (66.7–75.2)	162/456	35.5 (31.1–40.1)
UUI	Anterior	0	224/449	49.9 (45.2–54.6)	90/449	20.0 (16.4–24.1)
		1	169/298	56.7 (50.9–62.4)	85/298	28.5 (23.5–34.0)
		2	769/1161	66.2 (63.4–69.0)	313/1161	27.0 (25.8–32.1)
		3–4	554/842	65.8 (62.5–69.0)	243/842	28.9 (25.8–32.1)
	Posterior	0	470/709	66.3 (62.7–69.8)	212/709	29.9 (26.6–33.4)
		1	299/438	68.3 (63.7–72.6)	108/438	24.7 (20.7–29.0)
		2	697/1187	58.7 (55.9–61.5)	299/1187	25.2 (22.7–27.8)
		3–4	233/408	57.1 (52.1–62.0)	105/408	25.7 (21.6–30.3)
	Apex	0–1	826/1352	61.1 (58.4–63.7)	343/1352	25.4 (23.1–27.8)
		2	585/903	64.8 (61.6–67.9)	261/903	28.9 (26.0–32.0)
		3–4	278/463	60.0 (55.4–64.5)	114/463	24.6 (20.8–28.8)

CI, confidence interval; UUI, urgency urinary incontinence

^a^Answer ‘Yes’ in PFDI-20 questionnaire; ^b^bothersome symptom defined as answers 3: moderately and 4: quite a bit in PFDI-20 questionnaire (Scale = 0–4)
